# Wild Encounters: Analyzing Human–Animal Interactions in British and Irish Association of Zoos and Aquariums Facilities

**DOI:** 10.1002/zoo.70002

**Published:** 2025-07-07

**Authors:** Thomas Welsh, Emma L. Clayton, Annika Paukner, Ellen Williams, Samantha Ward

**Affiliations:** ^1^ School of Animal, Rural and Environmental Sciences Nottingham Trent University Southwell UK; ^2^ University Centre Askham Bryan York UK; ^3^ NTU Psychology Nottingham Trent University Nottingham UK; ^4^ Department of Animal Health, Behaviour and Welfare Harper Adams University Newport UK

## Abstract

Human–animal interactions (HAIs) are commonplace in zoos and aquariums, with a large proportion of these being animal–visitor interactions (AVIs). These AVIs range from visual contact through a barrier to direct physical contact in animal handling sessions. Due to the popularity of AVIs with a range of species, there is a need to understand what AVIs are occurring and to direct future animal welfare and visitor‐based research. The present study investigated the quantity and diversity of AVIs that occur in BIAZA‐accredited zoos and aquariums through a website review. The websites of full BIAZA members (*n* = 118) were assessed for opportunities where visitors interact with animals in an additional capacity, outside of interactions that form part of a traditional zoo visit. In total, 86% (*n* = 101) of members offered additional AVIs, with “meet and greet” (*n* = 389), “keeper for a day” (*n* = 137), and “walkthrough” (*n* = 96) being the most offered AVIs. Meet and greets were offered with 56 taxonomic families, and the mean cost and mean individual animals per organization were positively related to the number of meet and greets offered. Individual organization management style also had an impact on the model and should be an area for future study. Finally, a taxonomic bias was identified for meet and greets, as 71% (*n* = 41) of families were reported to be from the Mammalia class. This study provides a comprehensive evaluation of the quantity and quality of AVIs occurring in BIAZA organizations and highlights diversity in both species and types of interactions available to visitors. There is a need for further research on specific categories of AVIs most commonly seen: meet and greet, keeper for a day experiences, and walkthrough exhibits, as well as the impact of participating in the AVI from both animal welfare and visitor outcome perspectives.

## Summary

- Overall, 740 opportunities for animal–visitor interactions were identified.
- Meet and greets were the most offered interaction (*n* = 389), with 52 distinct taxonomic families.
- Number of individual animals, cost, and animal popularity were predictors of meet and greets being offered.

## Graphical abstract

We investigated the quantity and diversity of animal–visitor interactions in BIAZA‐accredited facilities, finding 740 opportunities for interactions across a range of contexts including walkthroughs and handling. Meet and greets were the most offered and were influenced by the number of individual animals, cost of interaction, and animal popularity.

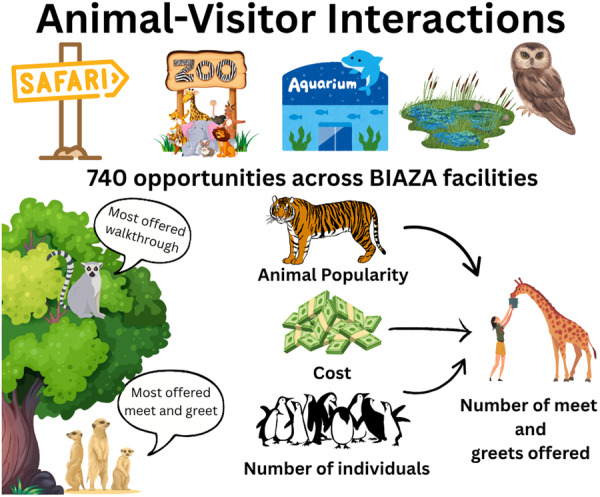

## 1 Introduction

Modern zoos and aquariums (hereby referred to as organizations) focus on five interconnected roles: conservation, education, research, animal welfare, and recreation (Fernandez et al. 2009). A priority for visitors is the opportunity to interact with animals, either visually, that is, through a barrier, or by having direct or indirect physical contact for an additional fee, through a specific animal experience (Godinez and Fernandez 2019; Campos et al. 2017). By offering these animal–visitor interactions (AVIs), organizations can increase their appeal, which in turn can lead to an increase in revenue and visitor satisfaction (Campos et al. 2017). As most organizations have charitable status, they rely on visitors as their main source of income. By offering AVIs at an added cost, additional income can be generated to maintain financial sustainability, which aids the organization in achieving its aims of conservation, education, and research. However, British and Irish Association of Zoos and Aquariums (BIAZA) guidelines state that AVIs should promote educational gain and inspire conservation‐related behaviors in visitors, rather than be purely for income generation (BIAZA 2023).

Education is one aim where AVIs can be used to improve visitor outcomes. Most education that occurs in a zoological organization is free‐choice learning, where visitors must have the motivation and interest to interact with the educational opportunities available (Tofield et al. 2003). Prior interest and an emotional connection to a topic are important factors in the effectiveness of free‐choice learning; therefore, these factors should be utilized to ensure informal education is effective (Clayton et al. 2017). Emotional connections can be influenced by an individual's predisposition to nature, their gender, and having a perceived up‐close encounter with an animal, in particular making eye contact (Powell and Bullock 2014). Allowing visitors to interact with animals provides multiple opportunities for emotional connections and a sense of attachment between a visitor and an individual animal that may lead to a desire to “save” that species from extinction (Myers et al. 2004). This desire can extrapolate to the species' habitat and promote proconservation behaviors, which helps organizations to meet their education goals (Clayton et al. 2009; Learmonth 2020).

D'cruze et al. (2019) conducted a global review of AVIs within World Association of Zoos and Aquariums (WAZA) members and found that 75% (*n* = 929) of organizations advertised at least one type of AVI, defining multiple types of AVIs including feeding, petting, riding, walkthrough, and show; highlighting the range of interaction opportunities being offered by organizations. “Petting areas” are typically an exhibit where domesticated or semi‐domesticated species, such as African pygmy goats (*Capra aegagrus hircus*), are allowed close direct contact with visitors and in some cases supervised feeding (Farrand et al. 2014; Anderson et al. 2002). Touch pools or tanks housing fish and aquatic invertebrates are similar to petting areas in that they allow for direct animal‐visitor contact (Biasetti et al. 2020). Touch tank interactions have been shown to stimulate social interactions between families and social groups (Kisiel et al. 2012) and have a positive impact on human mental wellbeing (Sahrmann et al. 2016). However, as described by Biasetti et al. (2020), there are multiple welfare and ethical implications from this AVI that should be managed effectively; primarily, species choice, enclosure size, and visitor supervision. Contrary to petting zoos and touch tanks, interactive talks and animal shows do not often include direct visitor contact with animals. This type of AVI is commonly seen in organizations and is generally viewed positively by the public (Fernandez et al. 2009). Animal shows are the third most popular AVI after animal petting and walk‐through exhibits, with an estimated 30% of WAZA organizations having a display or show (D'cruze et al. 2019). In the UK, these shows typically involve visitors sitting in an audience while animals exhibit trained behaviors, and animal‐related and conservation information is delivered by a staff member (Spooner, Jensen, et al. 2021). Walkthrough or immersive exhibits allow visitors unobstructed views of animals and allow visitors into the animal's enclosure. This type of exhibit often has minimal physical barriers between visitors and animals, and by putting the visitor into closer proximity to animals, evokes positive emotional responses which are linked to connectedness to wildlife and proconservation behaviors (Skibins and Powell 2013). Doodson et al. (2022) defined an additional type of AVI “meet and greet,” which focuses on “the opportunity for visitors to meet an animal under staff supervision, taking place inside the animal's enclosure or behind‐the‐scenes area; may involve physical contact, feeding or increased proximity to the animal; with or without a physical barrier present.” Meet and greets are opposed to “keeper for a day” experiences which are longer AVIs where the visitor adopts the role of a zookeeper; aiding with daily husbandry and often with multiple species (Martin and Melfi 2016). These AVIs outside of opportunistic interactions are a diverse and understudied area of zoo practice, which can have implications for both the animal and visitor involved.

It is likely that the distribution of AVIs within BIAZA members is different to those reported across the WAZA membership as described by D'cruze et al. (2019). It is important to recognize that this may be due to public perception of zoological organizations in the UK compared to other countries, which influences management practices; an example of this is that no dolphins are kept under human care in the UK, compared to 250 individuals in European Association of Zoos and Aquariums (EAZA) accredited facilities (Clegg et al. 2017). D'cruze et al. (2019) reported that for WAZA members, “Petting” comprised > 40% of advertised AVIs, and “Riding” was advertised more than expected, both of which are not commonplace across the UK and are discouraged under the Secretary of State's Standards of Modern Zoo Practice (SSSMZP) (Department for the Environment, Food and Rural Affairs 2012). It is important to understand the factors which influence the AVIs offered such as species holding and the cost of the AVI, not explored by D'cruze et al. (2019), as this can inform future priorities for AVI research. This will allow BIAZA organizations to design interactions which align with animal welfare goals and enhance visitor engagement and education outcomes.

The current study investigates the quantity and diversity of AVIs that occur in BIAZA‐accredited organizations in the UK and Ireland. Specifically, we aimed to identify the different types of AVIs; the quantity of each type and which taxonomic groups are being used most in AVIs, then assess factors influencing the most popular AVI being offered and suggest future directions for AVI research in organizations. We hypothesized that holding a higher number of individual animals and the animal being more popular with visitors could increase the number of AVIs being offered; whilst the more expensive the AVI, the fewer of these AVIs were offered.

## 2 Methods

### 2.1 Data Collection

BIAZA has 126 member organizations, split into full members (*n* = 120) and provisional members (*n* = 6) (BIAZA 2021). Only full members were included in the present study, which included organizations in the UK, Republic of Ireland, Gibraltar and Jersey.

Websites for the member organizations were found using the BIAZA “Find a member” search function (URL: https://biaza.org.uk/members/all). A total of 120 organization websites were used to collect data on the interactions being offered. During the data collection period, one zoo had closed, and another was due to close within 6 months; these were excluded from analysis. Each organization website was then systematically screened by a single individual (E. L. C) for information on the AVIs being offered by that organization. Information was noted including the type of organization (Table 1), if AVIs were offered, and the type and quantity of AVIs. All websites were reviewed between October 2021 and August 2022. Where information was available, AVIs were further categorized by the type of interaction (Table 2), the cost of the AVI, and the taxonomic family, or if the latter information was not available, the class of the species being used. It should be noted that due to this method, data regarding AVIs that were available at an organization, but not advertised on their website, were not collected. In this study, the term AVI follows the definition used by D'cruze et al. (2019) as “categories of activities that provide visitors (i.e., untrained nonstaff members of the public) with the opportunity to have indirect and direct contact with live captive wild animals (both inside and outside of their permanent enclosures)” (D'cruze et al. 2019, 2).

**Table 1:** Definitions of organization types.

Type	Definition
Zoo	An establishment which maintains a collection of wild animals that are primarily terrestrial, where visitors are required to walk around most of the time.
Safari park	An establishment which maintains a collection of wild animals that are primarily terrestrial, where visitors are required to drive around most of the time.
Aquarium	An establishment which maintains a collection of wild animals that are primarily aquatic or semiaquatic.
Specialist collection	An establishment which maintains a collection of wild animals that are from specific taxonomic class or order. For example, bird gardens or reptile parks.

**Table 2:** Definitions of animal–visitor interaction types.

Type of interaction	Definition
Petting area	Visitors enter an area of typically domesticated species and are allowed to directly interact with the animals within their enclosures, under staff supervision.
Animal handling	Direct interaction where visitors will normally hold or stroke/pet an animal under the supervision of a staff member. This is normally outside the animal's enclosure.
Meet and greet	Participants are offered the opportunity to meet specific animal species, under staff supervision. They take place inside the animal's enclosure or a behind‐the‐scenes area; may involve physical contact, feeding or increased proximity to the animal; with or without a physical barrier present; and last for under 2 h.
Keeper for a day	A longer interaction where a visitor will be accompanied by staff and will complete typical husbandry activities, as well as interacting with the animals as part of the experience.
Animal show	Interaction where an animal provides a demonstration of either natural or nonnatural behavior for visitors under the supervision of a staff member, with or without a physical barrier between them.
Walkthrough	Visitors will enter the animal's enclosure and have the opportunity for close proximity to the animal. There is no direct contact between the animal and visitor.
Driving	Visitors will enter the animal's enclosure in a vehicle and have the opportunity to be in close proximity to the animal, as part of an additional cost interaction. This interaction may include feeding or direct contact with the animal.

Species holding data per taxonomic family was compiled from the Species360 Zoological Information Management System (Species360 2022) detailing the number of individual animals and the number of BIAZA organizations that hold that taxonomic family. Family level was used over species level due to an assumption that in some cases a visitor would choose a meet and greet based on the taxonomic family rather than species, for example, penguins (*Spheniscidae*), and a lack of species‐level data on organization websites. To include the popularity of a family as a variable, the animal popularity index devised by Whitworth (2012) was used without modification, which was generated from a survey whereby UK participants were asked to rate specific animal characteristics that were then applied to animal groups, giving a popularity score for taxonomic groups found in BIAZA organizations. Where a specific taxonomic family did not feature in the animal popularity index, they were assigned a popularity score from the group that they were most morphologically similar to, as the animal popularity index was developed based on animal characteristics and typical behaviors (Whitworth 2012).

### 2.2 Data Analysis

All data analysis and visual presentation were completed using R Studio and R Statistical Software (v4.4.1, R Core Team 2021). A Fisher's exact test was used to determine if there was a significant association between the types of organization and how many interactions were offered. Meet and greets were identified as the most advertised AVI, which warranted further analysis. To determine factors which may influence the number of meet and greets offered per taxonomic family, a Gamma GLMM with log link function was used with the full data set due to the non‐normal distribution of the data and to ensure positive fitted values. The mean number of individuals per holding organization, mean cost, and animal popularity score were fixed effects, with the number of holding organizations a random effect. Further analysis for the remaining AVIs was not possible due to small sample sizes or limited species being used, therefore descriptive statistics are used to display the frequency and range of interaction opportunities being offered.

### 2.3 Ethics Statement

All data were publicly available and therefore, the ethics statement is not applicable.

## 3 Results

### 3.1 Summary of AVIs Across the BIAZA Membership

A total of 118 organizations were included in the analysis, of which 86% (*n* = 101) offered AVIs on their websites, while 14% (*n* = 17) did not. The Fisher's exact test (*p* < 0.05) indicated that there is a significant association between the type of organization and the number of AVIs advertised (Table 3). This suggests that zoos and safari parks are more likely to offer AVIs compared to aquariums and specialist collections.

**Table 3:** Number of BIAZA organizations which offer AVIs, split by organization type.

Type	Number of organizations	Offered interactions	No interactions
Zoo	81	91% (*n* = 73)	9% (*n* = 8)
Safari park	5	100% (*n* = 5)	0% (*n* = 0)
Aquarium	14	64% (*n* = 9)	36% (*n* = 5)
Specialist Collection	18	82% (*n* = 14)	18% (*n* = 4)
Total	118	86% (*n* = 101)	14% (*n* = 16)

**Figure 1:**
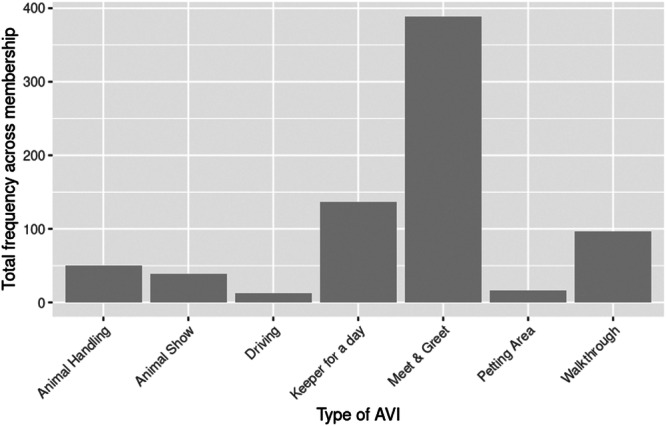
The total frequency of animal–visitor interactions being offered by BIAZA organizations.

Across the 101 organizations that offer interactions, 740 opportunities for AVIs were offered (mean AVIs per organization = 7.32) with meet and greet (*n* = 389), keeper for a day (*n* = 137), and walkthrough (*n* = 96) the most offered AVIs, while driving (*n* = 13) was offered the least (Figure 1).

**Figure 2:**
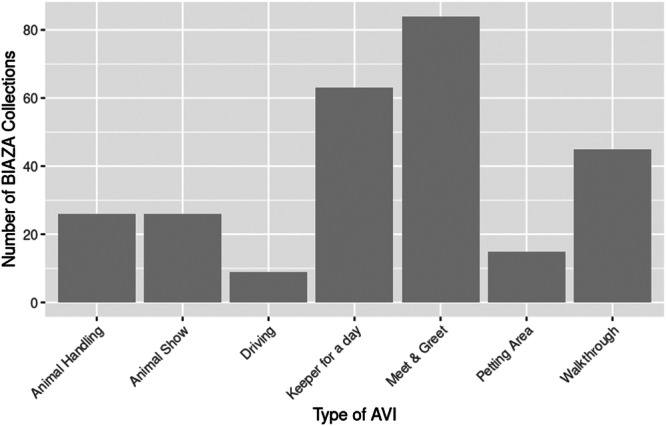
The number of BIAZA organizations offering each type of AVI.

The highest number of individual AVIs offered by an organization was 29, and a number of organizations offered only 1 AVI (*n* = 15). We found that the majority of organizations offered 2–10 AVIs (*n* = 60), some offered 11–20 (*n* = 24), and two offered 21–29. Most zoos (*n* = 81) offered a meet and greet interaction, while over half offered a keeper for a day (*n* = 63) (Figure 2).

### 3.2 Petting Areas, Animal Handling, and Animal Shows

**Figure 3:**
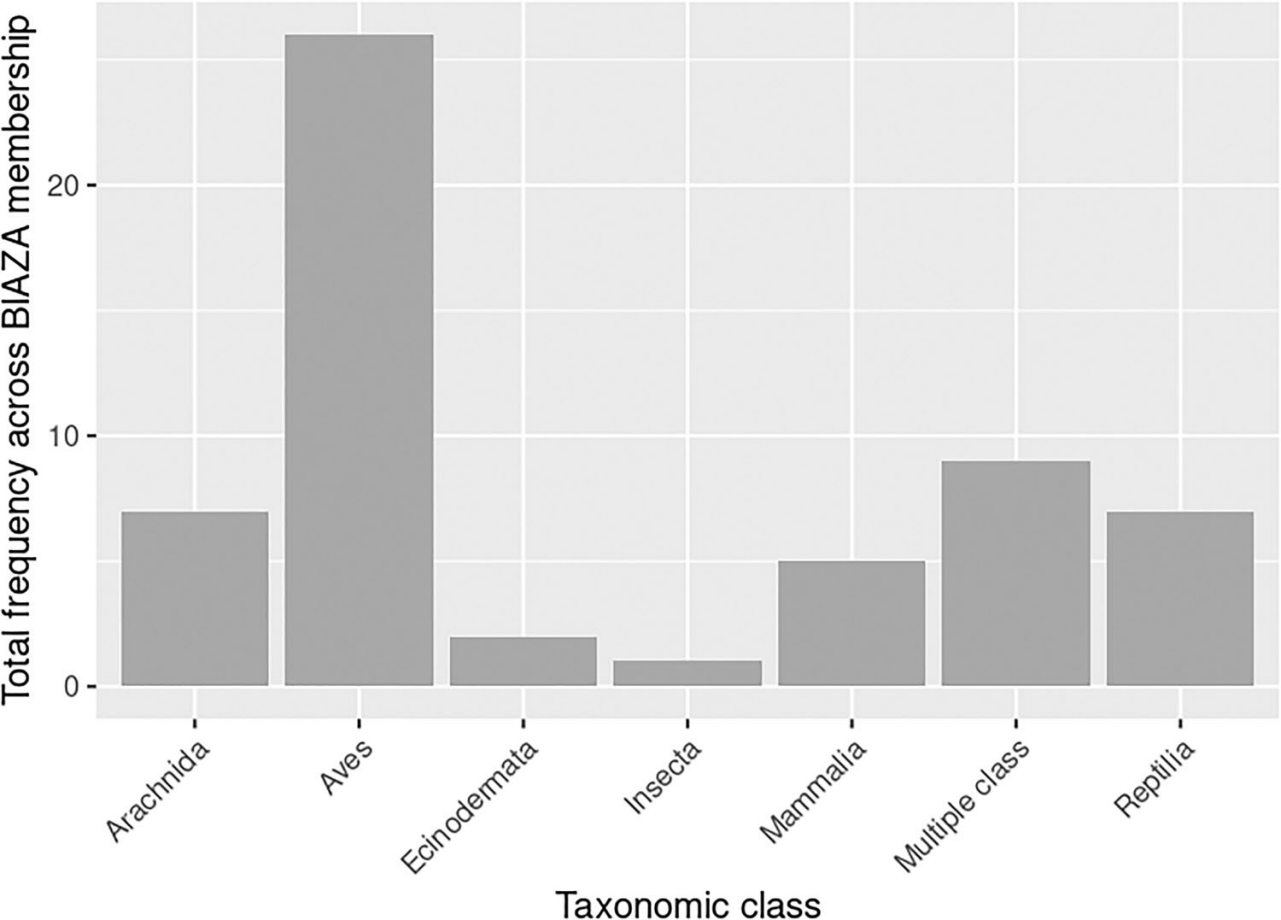
Total frequency of animal handling interactions offered across BIAZA organizations, split by taxonomic class.

In total, 15 organizations who offered AVIs included a petting area. However, compared to additional cost AVIs, no organization indicated an additional fee for this AVI. Information on the specific species being used in each petting area from websites was limited. Based on images on the organization website, this AVI was being offered using domestic animals including goats (*Capra hircus*), pigs (*Sus domesticus*), and rabbits (*Oryctolagus cuniculus*). A quarter of organizations (*n* = 26) who offered AVIs offered at least one animal handling interaction. Of these, 51 individual opportunities for animal handling were offered, and more than half (*n* = 26) included species from the class Aves, which were exclusively owls and birds of prey. Other animal handling AVIs consisted of species from the classes: Reptilia (*n* = 7), Arachnida (*n* = 7), Mammalia (*n* = 5), Insecta (*n* = 1), and Echinodermata (*n* = 2) (Figure 3). The remaining AVIs included species from several taxonomic classes being used in a single AVI. For example, one interaction named “Meet a Creature” included participants handling Madagascar hissing cockroaches (*Gromphadorhina portentosa*) and cornsnakes (*Pantherophis guttatus*) within the same animal handling activity.

A quarter of organizations (*n* = 26) who offered AVIs provided at least 1 animal show AVI, with 37 individual animal shows found across all BIAZA organizations. The most common animal show included solely birds of prey, accounting for 40% (*n* = 14) of all animal shows. This is followed by sea lions (Otariidae) (*n* = 10), mixed birds (*n* = 9), and shows containing animals from multiple taxonomic families (*n* = 4).

### 3.3 Meet and Greets

Meet and greets were the most common AVI, consisting of 51% (*n* = 389) of all AVI opportunities offered by BIAZA members. This type of AVI had the biggest range of species being used, with 13.4% (*n* = 52) of interactions including species from multiple taxonomic families within a single interaction. As meet and greets are additional cost AVIs (mean cost = £78, range = £5–£360), most specify a particular species the visitor will be given the opportunity to interact with. The family Herpestidae, which includes the meerkat *(Suricata suricatta)* were the most offered meet and greet encompassing 11.6% (*n* = 45), followed by the Felidae family with 11.1% (*n* = 42) and Lemuridae family at 9% (*n* = 35). Eighteen of the top 20 meet and greets species were from Mammalia, 1 was from Aves, and 1 was from Reptilia (Table 4).

**Table 4:** Number of individual meet and greets offered by BIAZA organizations, split by taxonomic family.

Family	Class	Number of meet and greets	Cost (£)	
			Mean	Range
Multiple taxa	N/A	52	80.50	10–260
Herpestidae	Mammalia	45	56	10–125
Felidae	Mammalia	43	126.79	45–310
Lemuridae	Mammalia	35	62	10–125
Giraffidae	Mammalia	23	94	10–210
Spheniscidae	Aves	23	71	10–110
Canidae	Mammalia	11	78	10–140
Mustelidae	Mammalia	11	54	26–75
Ailuridae	Mammalia	9	82	60–100
Rhinocerotidae	Mammalia	9	97	70–195
Testudinidae	Reptilia	9	62	30–85
Tapiridae	Mammalia	8	65	30–110
Bradypodidae	Mammalia	7	72	20–99
Dasypodidae	Mammalia	7	48	20–85
Otariidae	Mammalia	6	82	30–120
Caviidae	Mammalia	5	39	10–75
Hominidae	Mammalia	5	110	15–150
Macropodidae	Mammalia	5	70	50–95
Procyonidae	Mammalia	5	42	30.9–60
Ursidae	Mammalia	5	207	70–610
Elephantidae	Mammalia	4	150	60–250
Myrmecophagidae	Mammalia	4	61	30–80
Orycteropodidae	Mammalia	4	74	60–85
Strigidae	Aves	4	40	20–60
Bovidae	Mammalia	3	43	10–95
Equidae	Mammalia	3	60	40–95
Psittacidae	Aves	3	20	20
Varanidae	Reptilia	3	95	50–125
Callitrichidae	Mammalia	2	32.5	20–45
Camelidae	Mammalia	2	52.5	50–55
Carcharhinidae	Chondrichthyes	2	47.5	40–55
Cebidae	Mammalia	2	42.5	25–60
Cervidae	Mammalia	2	45	5–85
Cheloniidae	Reptilia	2	40	40
Pteropodidae	Mammalia	2	50	50
Suidae	Mammalia	2	60	45–75
Viverridae	Mammalia	2	22.5	10–35
Accipitridae	Aves	1	20	NA
Alligatoridae	Reptilia	1	20	NA
Apidae	Hymenoptera	1	80	NA
Atelidae	Mammalia	1	110	NA
Castoridae	Mammalia	1	70	NA
Cercopithecidae	Mammalia	1	85	NA
Chamaeleonidae	Reptilia	1	75	NA
Corvidae	Aves	1	54	NA
Crocodylidae	Reptilia	1	50	NA
Dasyatidae	Chondrichthyes	1	40	NA
Hyaenidae	Mammalia	1	85	NA
Hylobatidae	Mammalia	1	75	NA
Iguanidae	Reptilia	1	45	NA
Leporidae	Mammalia	1	75	NA
Mephitidae	Mammalia	1	40	NA
Petauridae	Mammalia	1	50	NA
Phascolarctidae	Mammalia	1	200	NA
Phocidae	Mammalia	1	60	NA
Pythonidae	Reptilia	1	20	NA
Tenrecidae	Mammalia	1	50	NA
Total/Range		389	78	5–365

**Figure 4:**
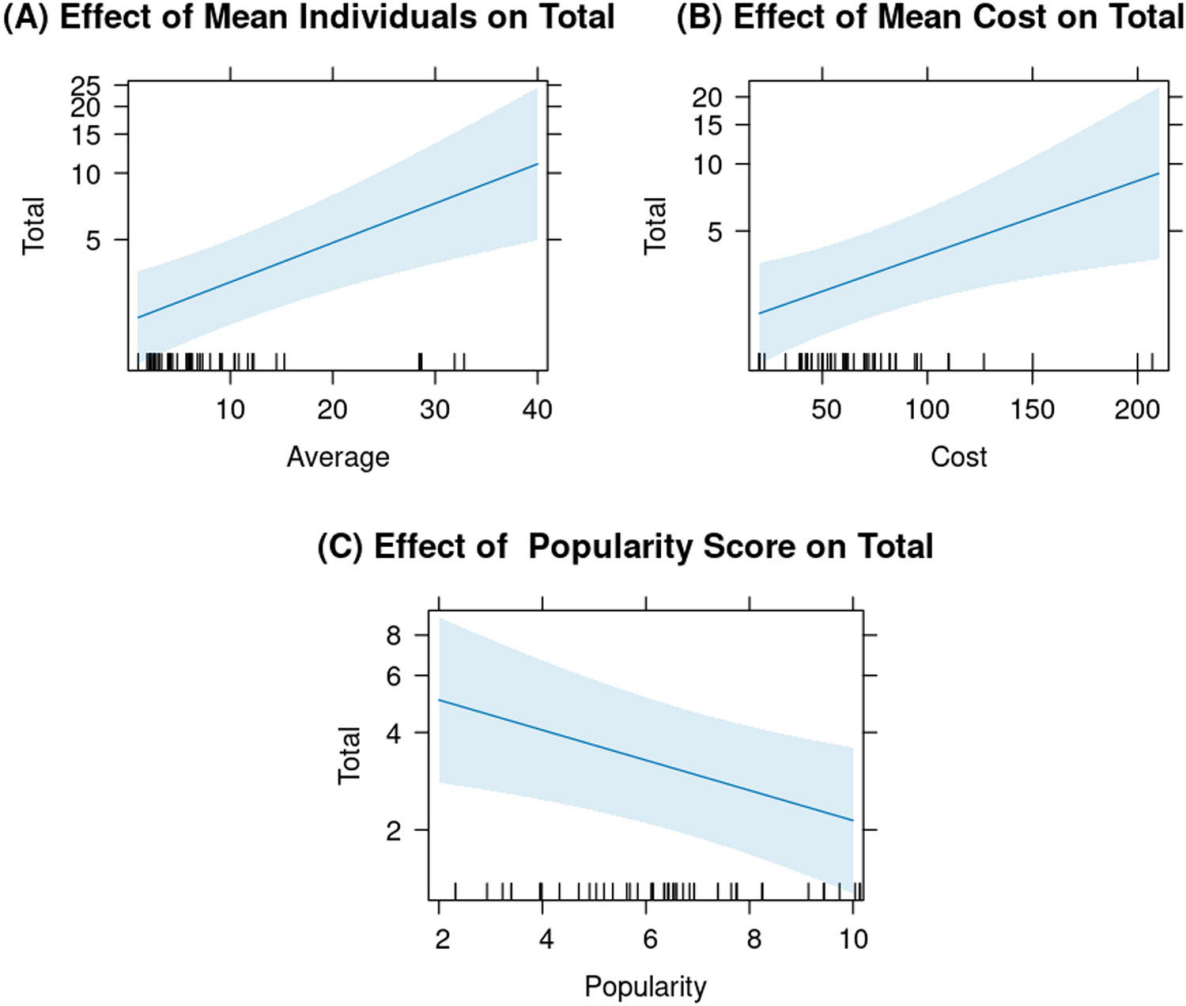
The effect of significant predictor variables on the number of meet and greets offered per taxonomic family. (A) Effect of mean individuals per organization. The regression line indicates a positive relationship between the mean number of individuals per organization and the total number of meet and greets offered. (B) Effect of mean cost. The regression line indicates a positive relationship between the mean cost and the total number of meet and greets offered. (C) Effect of popularity score. The regression line suggests a negative relationship between popularity score and total number of meet and greets offered. For all plots, the shaded area represents the 95% confidence interval.

The mean number of individuals per organization and the mean cost had significant positive effects on the quantity of meet and greets, meaning that organizations with a large number of individuals of a given species and charging a higher cost for a meet and greet were more likely to offer a meet and greet for that taxonomic family. Conversely, the animal popularity score had a significant negative effect on the quantity of meet and greets (Figure 4), meaning that organizations were less likely to offer meet and greets for highly popular animals. Residual variance for the random effect was higher than the variance, suggesting that the number of holding organizations represents a high amount of variability across different organizations (Table 5).

**Table 5:** GLMM of the total number of meet and greets offered per taxonomic family including AIC.

Model	Fixed effects	Estimate	SE	*t*	*p*	AIC
Full data set	Intercept	0.952	0.366	2.601	0.009	243.9
	Mean individuals of taxonomic family per organization^a^	0.041	0.011	3.781	< 0.001	
	Mean cost	0.008	0.003	2.792	0.005	
	Popularity score	−0.107	0.041	−2.621	0.008	
	*Random effect*	*Variance*	*SD*	*Residual variance*	*Residual SD*	
	Number of holding organizations	1.024	1.012	0.260	0.510	

a Mean individuals of taxonomic family per organization was calculated using Species360 data for the number of individuals in that taxonomic family held by BIAZA organizations, divided by the number of holding organizations.

**Figure 5:**
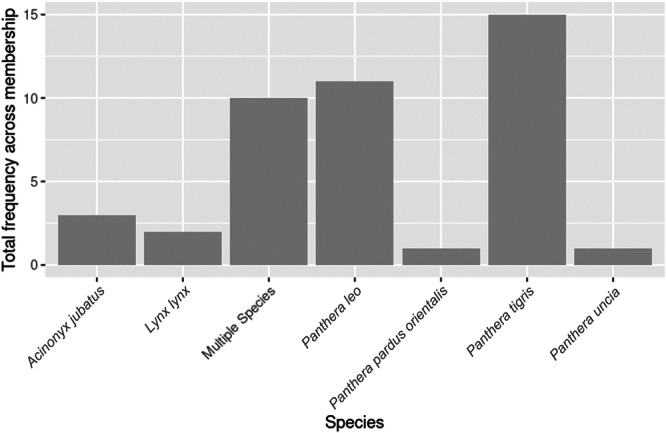
Frequency of meet and greets offered within the Felidae family across BIAZA organizations, split by species.

The Felidae family was the most diverse with meet and greets being offered for six individual species, with ten interactions featuring multiple species of Felidae (Figure 5).

### 3.4 Keeper for a Day, Walkthroughs, and Driving AVIs

**Figure 6:**
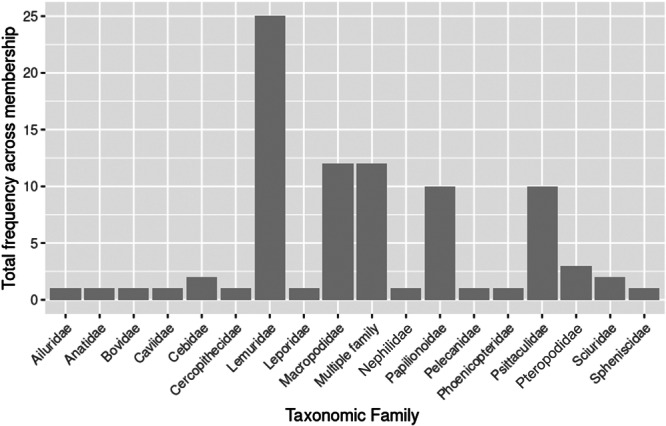
Total frequency of walkthrough interactions offered across BIAZA organizations, split by taxonomic family.

Keeper for a day experiences were the second most offered AVI, with 54% (*n* = 63) of all organizations advertising this type of interaction, and 137 individual keeper for a day experiences offered. The additional cost of these interactions ranged from £50 to £495 (mean cost = £174.10). Due to this type of AVI often including multiple species from a range of taxonomic groups, it was not possible to indicate specific species used for each experience due to the limited information provided on websites. Walkthrough exhibits were the third most offered interaction, with 96 walkthroughs in 45 BIAZA organizations. The greatest number of walkthrough exhibits in a single facility was 6, with 7 organizations having more than 3 walkthroughs. The remaining 38 organizations had fewer than 3 walkthroughs, and 72 organizations did not have any. Some exhibits housed species from multiple taxonomic families (*n* = 12) or mixed bird species (*n* = 9); however, where only one taxonomic family was housed, Lemuridae accounted for 26.3% (*n* = 25) of all walkthrough exhibits, followed by Macropodidae (*n* = 12) and Psittaculidae (*n* = 10) (Figure 6).

Nine organizations offered driving AVIs, with 13 AVIs across all BIAZA members. The AVIs were split between zoos (*n* = 4), safari parks (*n* = 4), and a specialist collection (*n* = 1). Most interactions included multiple species from the Mammalia class, with the exception of the interaction from the specialist collection, which included only Aves. The interactions noted here are additional cost interactions as opposed to those included within the entry fee, for example, using a minibus instead of a personal car for a drive‐through safari. The additional cost for these interactions ranged from £7 to £385 (mean = £92.88).

## 4 Discussion

Due to the range and quantity of AVIs offered, it can be inferred that AVIs are a popular element of a visit, with 740 individual AVIs being offered by BIAZA organizations. The percentage of organizations offering AVIs reported in the present study (86%) is higher than the 75% previously reported by D'cruze et al. (2019) who focused on WAZA members, reinforcing their finding that most organizations offer AVIs and that there is a need for further study of the impact of these on animal welfare, visitor education, and support for conservation. The higher percentage of organizations offering AVIs within BIAZA compared to the wider WAZA membership suggests that despite more stringent animal welfare legislation, cultural differences on the use of animals, and public perception of the role of zoos, the UK and Irish public still wish to interact with zoo animals when offered by accredited organizations (Bacon et al. 2021; Lee 2015). Most organizations within BIAZA offered AVIs, with all five safari parks offering AVIs. Aquariums offered the least amount of AVIs, with 71% not offering any AVIs; this may be due to housing aquatic species which are more difficult for visitors to interact with, outside of touch‐pools and swim interactions (Biasetti et al. 2020). Diving is considered a high‐risk activity that carries additional health and safety considerations, maintenance of equipment and requires suitably qualified staff which could discourage organizations from offering this type of AVI (BIAZA 2020). Although not directly comparable with the results presented here, D'cruze et al. (2019) reported that the “drive through/cage dive” and “walk or swim through” types of AVI were advertised more in European organizations compared to other regions, which was not seen in the present study. Zoos were the largest category of organization identified, with 91% offering interactions. Most organizations which offered interactions advertised between 2 and 10 individual AVIs, suggesting that they recognize the importance of AVIs for visitor satisfaction, education, and income generation. However, this should be balanced to avoid potential negative visitor and animal outcomes (Doodson et al. 2022; Learmonth 2020).

Compared to other AVI types, petting areas and animal handling AVIs were less commonly seen across BIAZA organizations, although this may be due to not being advertised on an organization's website. This finding contrasts with D'cruze et al. (2019), who reported “petting” as the most prevalent AVI type advertised across the WAZA membership. This difference could be due to the more stringent legislation in the UK/Ireland and BIAZA policies that need to be adhered to compared to other countries, such as the BIAZA Close Contact Policy (BIAZA 2023), and although there are WAZA guidelines on AVIs, these are less stringent than the policies set out by BIAZA (WAZA 2021). In addition, there are potential biosecurity and zoonotic disease transmission risks, such as transmission of *Escherichia coli, Salmonella*, and *Campylobacter* which may dissuade organizations from offering these types of AVI (Weese et al. 2007). Birds (Aves) were the most common class of animal used in animal handling AVIs, predominantly due to specialist collections housing mainly birds of prey. Most animals housed in these organizations will be trained for handling and habituated to human contact as a key part of their husbandry and management (Parry‐Jones 2001). Reptiles (Reptilia) and insects (Insecta) were also used in animal handling, perhaps due to their smaller size and ability to be removed from their enclosure for visitor handling. Spiders (Arachnida) were used in animal handling and could be considered an educational tool to assist in the redirection of negative connotations toward these species, which humans typically associate with fear and danger (Prokop and Fančovičová 2013). Animal shows reported here (22% of all organizations, *n* = 26) are fewer than the 30% of WAZA members reported by D'cruze et al. (2019) that offer this AVI type. This may be due to a lower number of marine mammals held in UK/Irish organizations; for example, there are no cetaceans held in BIAZA organizations.

Meet and greets were the most offered interaction across all organizations that offered AVIs (*n* = 389) with 81 organizations offering this AVI type, consistent with findings by Doodson et al. (2022). Most meet and greets are typically lasting 5 min to 1 h (Spooner, Farnworth, et al. 2021); which allow organizations to offer more of these interactions compared to keeper for a day, which is typically an hour or longer experience. Within meet and greets, there is a taxonomic bias present with most interactions reported as being with the class Mammalia. This could be due to visitor interest in mammals, which may have influenced organization planning prioritizing these species (Moss and Esson 2010; Brereton and Brereton 2020). For example, Albert et al. (2018) analyzed the 20 most charismatic species based on surveys; 14 taxa also feature within the meet and greets offered by BIAZA organizations, which could contribute to the large quantity of meet and greets seen with mammals. Mean cost of a meet and greet was a significant positive predictor of the number of meet and greets offered, suggesting that visitors are more likely to pay a premium for an AVI with an animal that is seen as charismatic; with the mean cost of a meet and greet with a mammal being generally higher than other classes (Albert et al. 2018; Powell and Bullock 2014). The most expensive individual meet and greet was with polar bears *(Ursus maritimus)* costing £610, although this was the same price for one or two visitors.

The family Herpestidae, which included predominantly the meerkat, was the most frequent meet and greet. This species is commonly held in BIAZA organizations (individuals *n* = 570, holding organizations *n* = 73), is considered to be popular with visitors, and is often used as an ambassador species (Williams et al. 2021). The popularity of this species could also be attributed to the “Compare the Meerkat” advertising series, which anecdotally has led to increased visitation of meerkat enclosures (Hearn 2009). Although animal popularity score had a significant negative effect on the total number of meet and greets offered with a family, this result should be taken with caution due to the methods used to determine the animal popularity index developed by Whitworth (2012). The methods used to determine characteristics considered popular with the public and then assigned these to zoo species to generate a popularity score; rather than asking the public directly which species they prefer. However, research on animal popularity with visitors in zoological organizations is lacking and restricted to case studies (e.g., Carr 2016), which is why the popularity score index was deemed the most appropriate measure available for the current study. Using the popularity score index, there are a range of primate families within the data set which have high popularity scores but low numbers of meet and greets offered. For example, gibbons (Hylobatidae) had the highest popularity score but only one meet and greet across BIAZA organizations. The lack of meet and greets with primate families, excluding Lemuridae, may be due to additional health and safety considerations associated with meet and greets for category one species (BIAZA 2023). Another possible factor is biosecurity and zoonotic disease transmission, such as SARS‐CoV‐2 (Dusseldorp et al. 2023) between visitors and primates which is less of a concern for other species. The number of holding collections for families with a high popularity score may also influence the number of meet and greets offered, for example, only 30 BIAZA organizations hold gibbons and therefore, it is unlikely that a high number of meet and greets would be seen.

Although the popularity score has a negative effect on the number of meet and greets offered, overall there was a large quantity and diversity of meet and greets that included species from 56 taxonomic families. The diversity of species compared to other types of AVI and high variance could be attributed to the management styles of individual organizations, where positive reinforcement training can be used to facilitate meet and greets with visitors through protected contact (a physical barrier separating the animal and human). This allows a safe environment for the visitor whilst still able to interact with the animals. The Felidae family was the second most offered taxonomic family for meet and greets and most diverse where species‐level data were available. The welfare of felids that participate in AVIs is an understudied area. However, research that has been conducted report mixed effects such as no changes in fecal‐glucocorticoid metabolites in serval (*Leptailurus serval*) (Acaralp‐Rehnberg et al. 2020) and an increase in pacing behavior of lions (*Panthera leo*) and tigers (*Panthera tigris*) (Szokalski et al. 2013). However, AVIs that include felids typically involved trained animals, so further research is needed to determine the effect of the AVI itself, as other training‐related elements may be influencing the results presented such as food‐anticipatory behavior, as interpreted by Szokalski et al. (2013).

After meet and greets, the keeper for a day experience was the second most offered AVI across BIAZA organizations. Keeper for a day typically operate by the visitor shadowing a keeper as they complete their daily routine (Martin and Melfi 2016). Contact with keeping staff and animal interactions are considered important factors in education outcomes for visitors, so it could be considered a highly educational experience for the participant (Godinez and Fernandez 2019; Skibins and Powell 2013). There are also various opportunities for emotional connections to be formed through being in close contact with the animals, which can be extrapolated to concern for the environment and conservation mindedness (Powell and Bullock 2014).

Walkthrough exhibits were the third most offered AVI, both in terms of the number of organizations and the frequency of AVI. This type of interaction is increasing in popularity due to being able to engage multiple senses in the visitor to improve free‐choice learning, connectedness to nature, and concern for a species' wild counterparts (Chih Mun et al. 2013). They also allow unstructured interactions to occur between animals and visitors with minimal physical barriers, which can influence a visitor's emotional responses and promote learning (Pollastri et al. 2022). Benefits to visitor wellbeing of being in walkthroughs are now being suggested, with research in this area increasing (Sumner and Goodenough 2020). Species from the Lemuridae family accounted for over a quarter of walkthrough exhibits, this may be due to the family being a popular species held in BIAZA organizations and their popularity with the public due to their perceived charisma, aesthetic appeal, and friendliness (Howell et al. 2019). From an operational perspective, visitors perceive animals in walkthrough exhibits to have improved welfare and higher educational value than traditional zoo enclosures (Price et al. 1994). In addition, there is evidence that this family, in particular ring‐tailed lemurs (*Lemur catta)* are suitable for this type of AVI. For example, ring‐tailed lemurs rarely react to visitors during potential interactions and visitor variables generally have a limited impact on lemur behavior (Collins et al. 2017). Instead, other factors have a greater impact on lemur behavior, such as weather (Collins et al. 2017; Goodenough et al. 2019; Farhall and Litten‐Brown 2010). A recent meta‐analysis by Hosey et al. (2023) using existing studies suggests that walkthrough and semi‐free range enclosures are associated with more neutral/positive responses from primates, although this should be interpreted with caution due to the low number of studies in this area. However, it is important to note the variability in enclosure design, visitor management, and previous experiences of the individual animals, which will also influence the impact that visitors will have within walkthrough exhibits (Pollastri et al. 2022).

Guidelines on the operation of AVIs have been provided by the WAZA (2021), as well as regional organizations including the Association of Zoos and Aquariums (AZA), BIAZA, and the EAZA. These focus on the suitability of a species for interactions, health and safety for visitors, educational outputs, and animal welfare monitoring. However, the impacts of these are not assessed on a regular basis and can be difficult due to limited research on specific species and types of interactions (Spooner, Farnworth, et al. 2021; D'cruze et al. 2019). Due to the vast range of AVIs occurring, further research is required on the effects of these interactions (both during and following the experience) on both the visitor and individual animals involved, in particular meet and greets, keeper for a day, and walkthrough exhibits which are the types of AVI most commonly seen in BIAZA organizations.

## 5 Conclusion

There were a wide range of AVIs being offered across BIAZA organizations. These AVIs ranged from short‐term meet and greets and walkthrough exhibits, to longer‐term keeper for a day experiences as well as novel driving interactions. A high percentage (86%) of BIAZA organizations offered AVIs, therefore it is clear they are a key part of modern zoological organizations' operations and could have a multitude of benefits for both the organization and their visitors. However, there are welfare implications and ethical considerations that must be monitored, evaluated, and managed accordingly to ensure high animal welfare standards and visitor outcomes are met. This study has demonstrated the types of AVIs occurring within BIAZA organizations, and the quantity and diversity of species being used in those AVIs. For meet and greets, mean cost, animal popularity, and mean number of individual animals per organization have been identified as significant predictors of the number of meet and greets being offered. However, the variability between organizations suggests additional research into the impact of zoo management style and perception of zoo management regarding AVIs would be beneficial, as would further situation‐specific research on AVIs and their implications for both visitor and animal outcomes.

This study provides an evaluation of the quantity of AVIs occurring in BIAZA organizations and highlights diversity in both species chosen for interactions and types of interactions available to visitors. We encourage further research on specific categories of AVIs most commonly seen: meet and greet, keeper for a day experiences, and walkthrough exhibits, as well as the impact of participating in the AVI from both animal welfare and visitor outcome perspectives.

## Ethics Statement

All data were publicly available and therefore, the ethics statement is not applicable.

## Data Availability

The data that support the findings of this study are available from the corresponding author upon reasonable request.
